# Supplementary material for: Engineered hypermutation adapts cyanobacterial photosynthesis to combined high light and high temperature stress
Source: Nat Commun. 2023 Mar 4;14:1238. doi: 10.1038/s41467-023-36964-5 (PMC9985602; doi:10.1038/s41467-023-36964-5)
Supplement: Supplementary file 1 — Supplementary Information [file 41467_2023_36964_MOESM1_ESM.pdf]

Engineered hypermutation acclimates cyanobacterial photosynthesis to high light and high temperature stress

Sun and Luan *et al.*

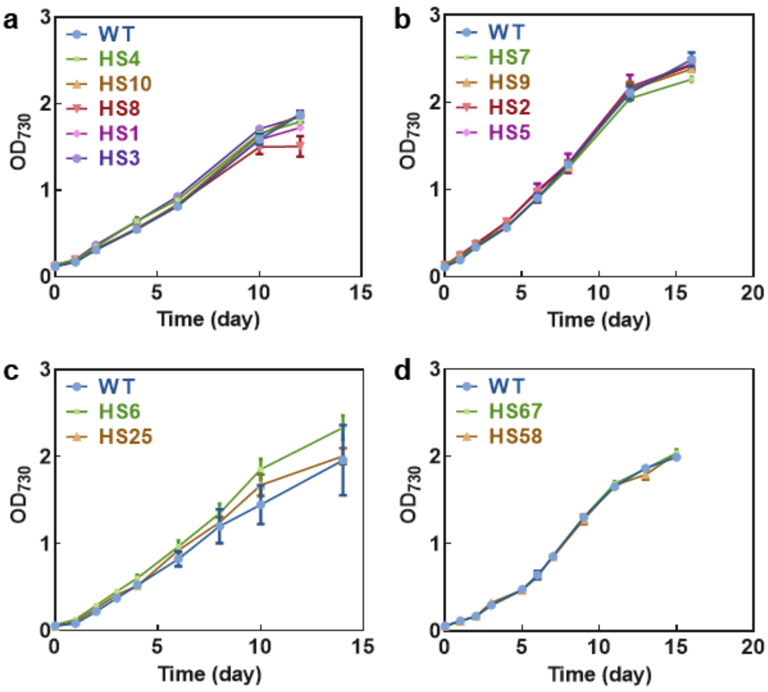

Supplementary Figure 1. The growth curves of *Synechococcus* mutants with disturbed DNA replication fidelity machinery. The growth of *Synechococcus* mutants carrying a single inactivated/overexpressed fidelity-related gene and the wildtype control were evaluated at 30°C and 50 μmol photons/m^2/s (white fluorescent light). Shown were the growth curves of HS4 (△*dam*::Kan^r), HS10 (△*mutS*::Sp^r), HS8 (△NS1::Kan^r-$P_{cpcB560}$-*recN*), HS1 (△*uvrB*::Kan^r), HS3 (△*xthA*::Kan^r) in (**a**), HS7 (△NS1::Kan^r-$P_{cpcB560}$-*recA*), HS9 (△NS1::Kan^r-$P_{cpcB560}$-*umuC*), HS2 (△*uvrC*::Kan^r), HS5 (△*mutY*::Kan^r) in (**b**), HS6 (△*mutM*::Kan^r), HS25 (△NS2::Sp^r-$P_{cpcB560}$-*umuD*-*umuC*) in (**c**), and HS67 (△*mutL*::Ge^r), HS58 (△NS1::Km^r-$P_{cpcB560}$-*umuD*) in (**d**). NS1 refers to neutral site I and NS2 refers to neutral site II (The same below). The experiment was replicated more than twice to ensure its reliabilities. Data are presented as mean values ± SD (n = 3 biological replicates). Source data are provided as a Source Data file.

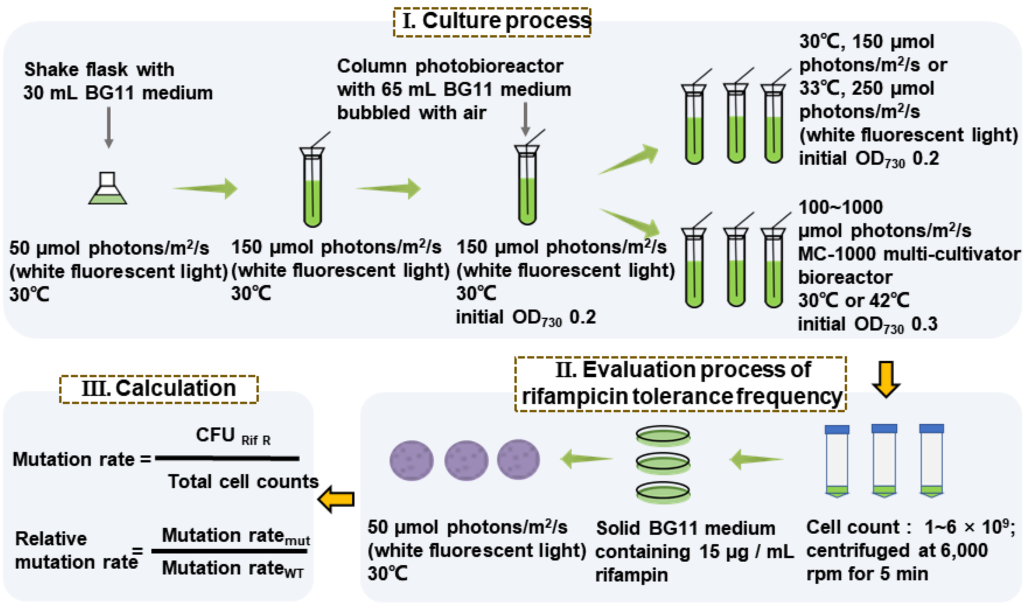

Supplementary Figure 2. Schematic evaluation process for replication mutation rate. The *Synechococcus* strains would first be inoculated into 30 mL BG11 medium shaking under 50 μmol photons/m^2/s white fluorescent light at 30°C. After reaching the exponential growth period, the *Synechococcus* cells would be pre-cultivated in BG11 medium with an initial OD_{730} of 0.2 in column photobioreactors and cultivated under 150 μmol photons/m^2/s white fluorescent light at 30°C, bubbled with air for two rounds. Then, the pre-cultivation broth would be re-inoculated into fresh BG11 medium and cultivated under the same conditions to the mid-exponential phase for general evaluation process, or cultivated under increased temperature and light intensity conditions as necessary for hypermutation evolution process. After that, approximately 1×10^9 to 6×10^9 cells would be collected and plated onto a solid BG11 medium containing 15 μg/mL rifampin. The rifampin-resistant colonies would be counted after two weeks of cultivation, and frequencies of the resistant cells in the original culture broth would be calculated to evaluate the mutation rates of the *Synechococcus* genome replication. The relative mutation rates of the recombinant strains would be calculated by comparing with that of the wildtype control.

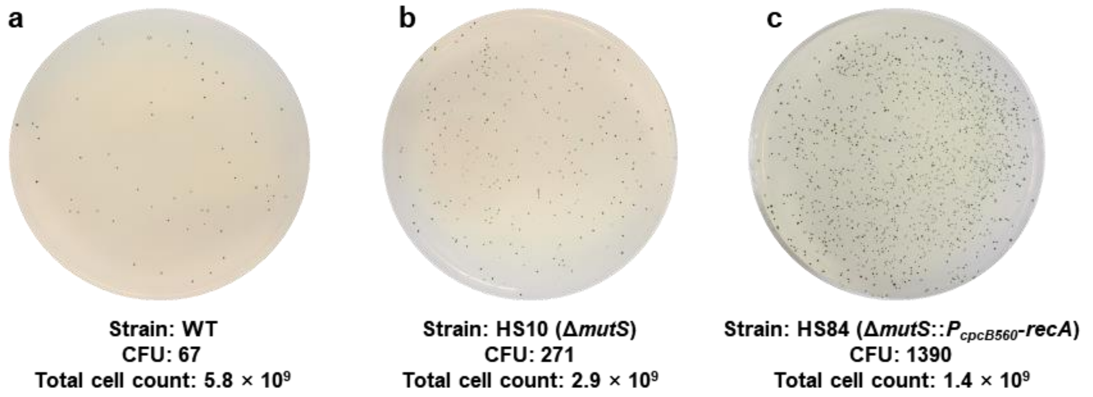

Supplementary Figure 3. Photographs of representative rifampicin-tolerant colonies in the general mutation rate evaluation processes. After cultivated under 150 μmol photons/m^2/s white fluorescent light at 30°C to mid-exponential phase, approximately 1 × 10^9 to 6 × 10^9 cells would be collected and plated onto a solid BG11 medium containing 15 μg/mL rifampin. The rifampin-resistant colonies would be counted after two weeks of cultivation. Shown here are the photographs of the solid BG11 medium containing 15 μg/mL rifampin from the general evaluation of mutation rates of WT (**a**), HS10 (**b**), and HS84 (**c**) strains. The cell numbers were calculated by an automated cell counter and the CFU would be counted using Image J.

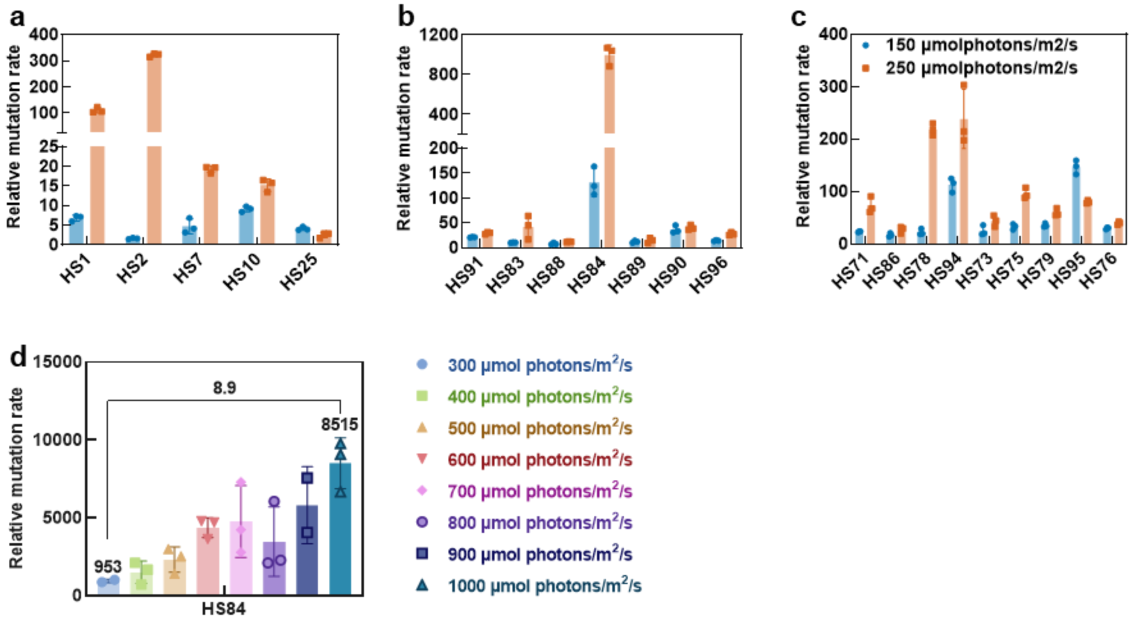

Supplementary Figure 4. The genome replication mutation rates of the recombinant strains with engineered DNA replication fidelity machineries were affected by the cultivation conditions. The relative mutation rates of *Synechococcus* mutants carrying a single (**a**), or two (**b**), or multiple (**c**) inactivated/overexpressed fidelity-related genes under 30°C and 33°C, with the light intensities of 150 and 250 μmol photons/m^2/s. The relative mutation rate data for some special batch of HS84 strain (Δ*mutS*::$P_{cpcB560}$-*recA*) cultivated under 42°C with the light intensities of 300, 400, 500, 600, 700, 800, 900, and 1000 μmol photons/m^2/s (**d**). Data are presented as mean values ± SD (n = 3 biological replicates except the first and 7th column of **d** which includes two biological replicates). The experiment was replicated more than twice to ensure its reliabilities for **a**, **b**, and **c**. Data in **d** were obtained from some special batch. Source data are provided as a Source Data file.

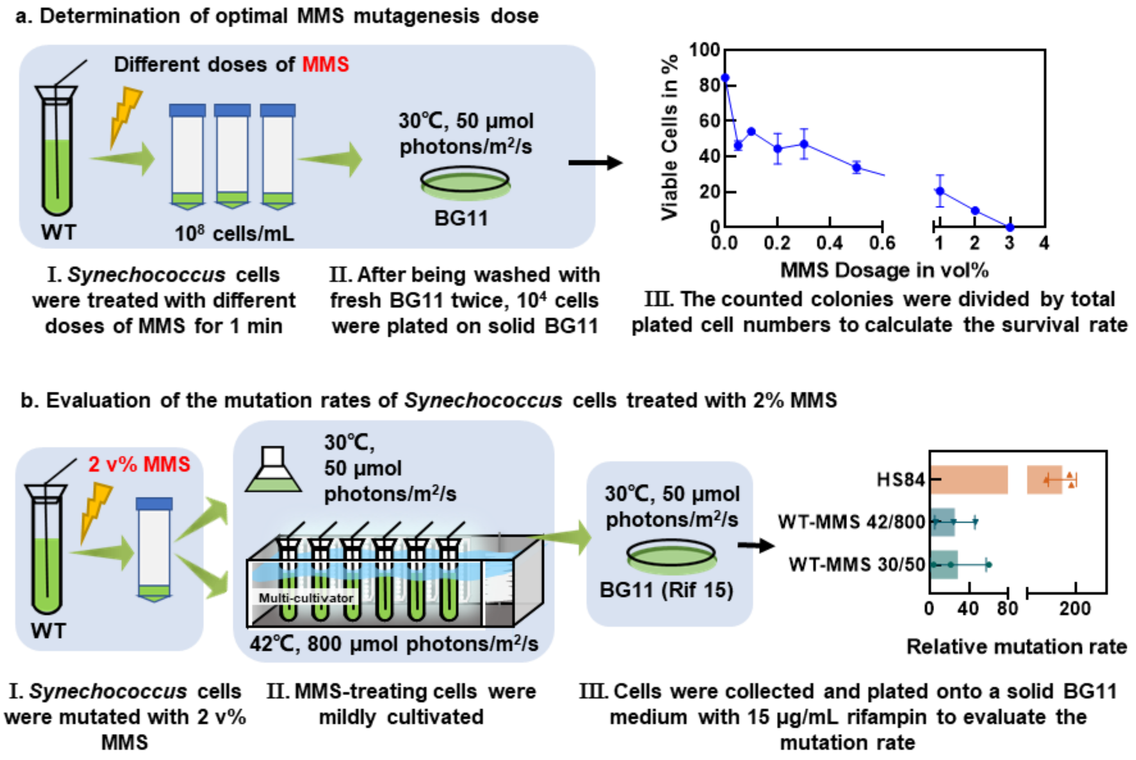

Supplementary Figure 5. Determination of the lethality and mutagenetic effects of MMS. a. WT cells with densities of 1 x 10^8 cells/mL were incubated with up to 3% MMS for 1 minute. Cells were then washed with fresh BG11 twice and 10^4 cells were plated on solid BG11. The counted colonies were divided by total plated cell numbers to calculate the survival rate. b. WT cells with a density of 10^8 cells/mL was incubated with 2% MMS for 1 minute. MMS-treated cells were then washed with fresh BG11 twice and then inoculated in BG11 medium at an initial OD_{730} of 0.2 in a flask under 50 μmol photons/m^2/s white fluorescent light at 30°C or in column photobioreactors and cultivated in MC1000 under 42°C and 800 μmol photons/m^2/s (as used to elevate mutation rates of HS84). Approximately 1×10^9 to 4×10^9 cells were collected and plated onto a solid BG11 medium containing 15 μg/mL rifampin. The rifampin-resistant colonies were counted after two weeks of cultivation to calculate the mutation rate (WT-MMS 30/50 or WT-MMS 42/800). Mutation rates of non-treated WT cells and HS84 cells (as described in the manuscript) would be calculated as controls. Data are presented as mean values ± SD (n = 3 biological replicates). The experiment was replicated more than twice to ensure its reliabilities. Source data are provided as a Source Data file.

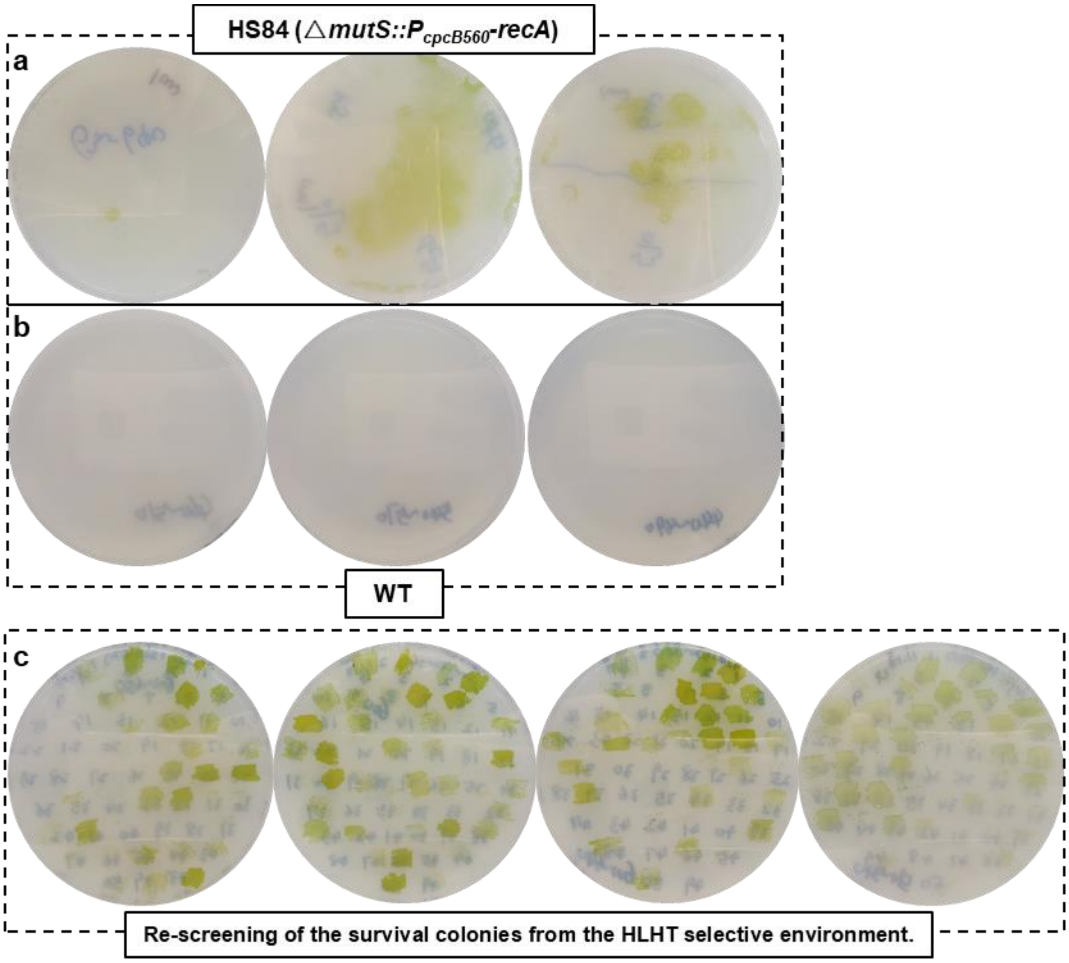

Supplementary Figure 6. Phenotype screening results of hypermutable strains for improved high temperature and high light tolerance. After the mutagenesis culture step, 10^9 cells in the logarithmic growth phase were harvested and coated on BG11 plates and cultivated in an incubator at conditions of 44°C and 500 μmol photons/m^2/s (warm white light) for screening. After 4 days of cultivation, hundreds of HS84 colonies were obtained (**a**), while no wildtype *Synechococcus* cells could survive (**b**) in the selective environment. Then, the survival colonies from the screening step were stretched on fresh BG11 plates for re-screening in the incubator under the same conditions (44°C and 500 μmol photons/m^2/s). After 4 days of cultivation, approximately 180 out of 200 colonies could still survive the screening conditions (**c**). The experiment was replicated more than twice to ensure its reliabilities.

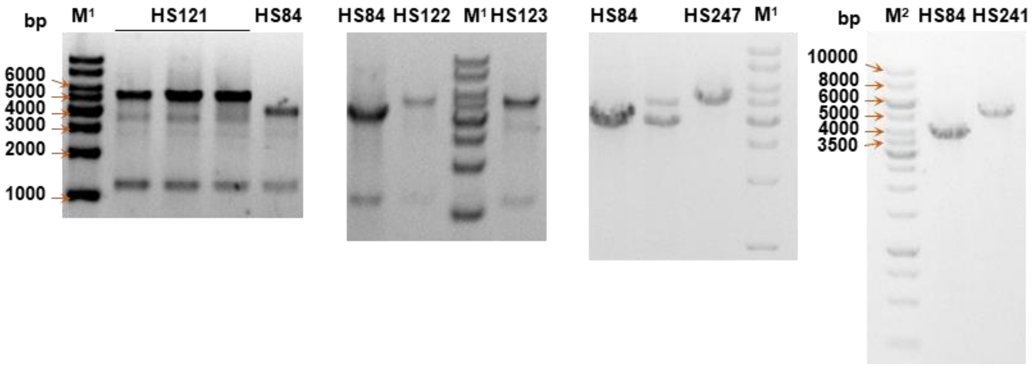

Supplementary Figure 7. Genotype identification of the strains that were complemented by restoring the *mutS* gene and removing the additional *recA* using gel electrophoresis. HS121, HS122, HS123, HS247, and HS241 were obtained from the transformation of HLHT-tolerant evolved strains (Δ*mutS*::$P_{cpcB560}$-*recA*) with pHS81. The original strain of HLHT-tolerant evolved strains was HS84 (Δ*mutS*::$P_{cpcB560}$-*recA*). The primers used here were PUC19-mutSUP500-F and mutSDOWN500-puc19-R listed in Table S4. The expected size of HS121, HS122, HS123, HS247, and HS247 was 5.343 Kb, and that of HS84 was 4.161 Kb. M^1 was 1 Kb DNA Ladder (TransGen, Beijing, China) and M^2 was GeneRuler 1Kb DNA Ladder (Thermo Fisher, US). The experiment was replicated more than twice to ensure its reliabilities.

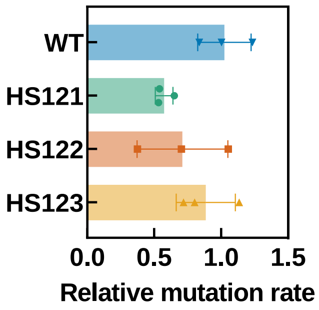

Supplementary Figure 8. Relative replication mutation rates of the three evolved *Synechococcus* strains. Data are presented as mean values ± SD (n = 3 biological replicates). The experiment was replicated more than twice to ensure its reliabilities. Source data are provided as a Source Data file.

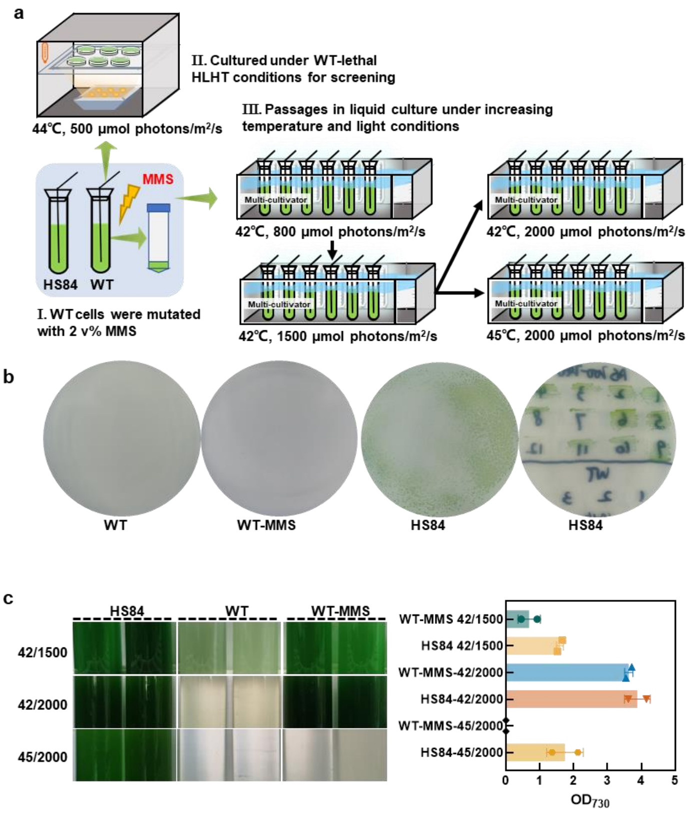

Supplementary Figure 9. Comparison of the engineered hypermutation system and MMS-induced random mutagenesis method on improving HLHT tolerance of *Synechococcus*. a, Diagram for the process of evolving HLHT tolerance with MMS-treated WT cells and hypermutable HS84 cells. The *Synechococcus* cells were inoculated in BG11 medium at an initial OD_{730} of 0.2 in column photobioreactors and cultivated under 150 μmol photons/m^2/s white fluorescent light at 30°C. WT cells (10^8 cells/mL) were incubated with 2% MMS for 1 minute. Cells were then washed with fresh BG11 twice. And then approximately 1×10^9 cells of HS84, WT, and WT treated with MMS were collected and plated onto solid BG11 medium and cultured under WT-lethal HLHT conditions (44°C and 500 μmol photons/m^2/s, warm white light). Meanwhile, HS84, WT, and WT treated with MMS were inoculated in BG11 medium at an initial OD_{730} of 0.2 in column photobioreactors and cultivated in MC1000 under 42°C and 800 μmol photons/m^2/s. Then, cells cultured in the mid-log phase were inoculated in BG11 medium at an initial OD_{730} of 0.05 in column photobioreactors and cultivated in MC1000 under 42°C and 1500 μmol photons/m^2/s. After that, cells cultured in the mid-log phase were inoculated in BG11 medium at an initial OD_{730} of 0.05 in column photobioreactors and cultivated in MC1000 under 42°C and 2000 μmol photons/m^2/s and 45°C and 2000 μmol photons/m^2/s. **b,** HLHT tolerance screening results of MMS-induced WT and HS84 on solid plates. **c,** Results of the evolution of HLHT tolerance through passages in liquid cultivation. Data are presented as mean values ± SD (n = 2 biological replicates). More than two independent replications of this experiment were performed to ensure the reliabilities. Source data are provided as a Source Data file.

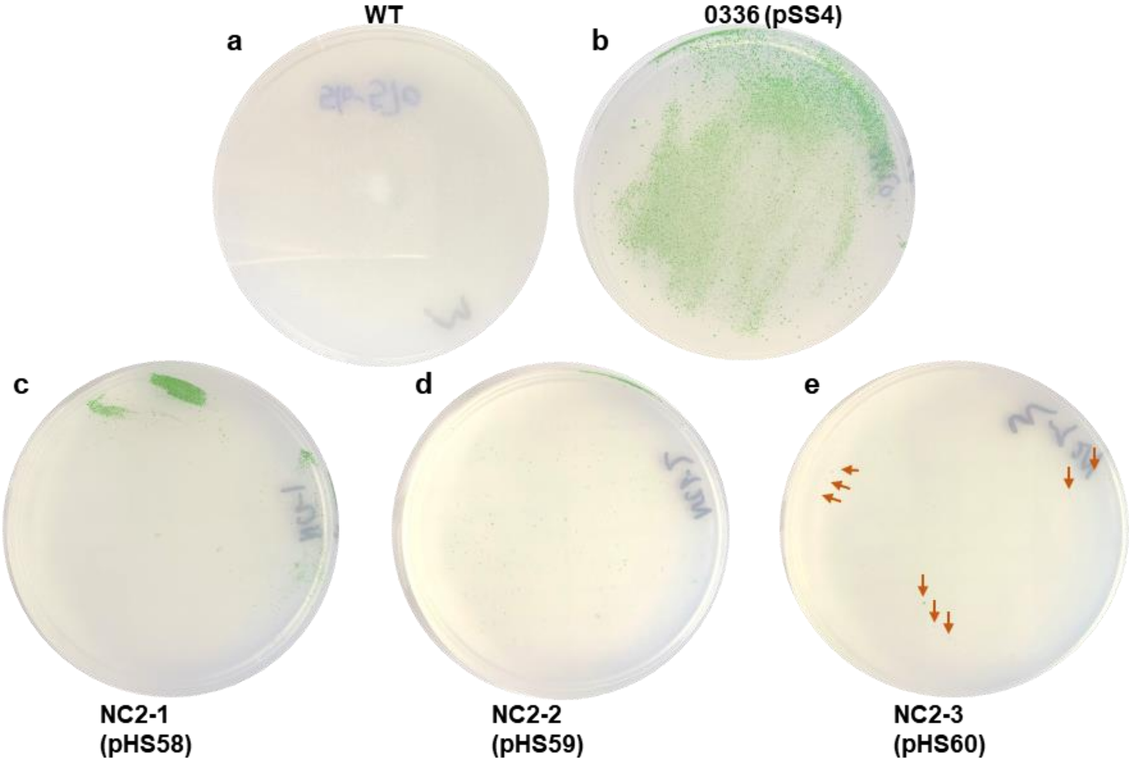

Supplementary Figure 10. Obtaining of positive transformants tolerant to high temperature and high light conditions. For strategy I in Fig 5a, homologous fragments containing mutations (NC2-1, NC2-2, NC2-3, NC3-1, 0884, 1799, 1977, 1831-1, 0189-1, 2215-2) were assembled in plasmids and transformed into *Synechococcus* cells, while the plasmid containing fragments with 0336 mutation was used as a positive control, which would be screened under WT-lethal light intensity and temperature. And WT was cultured under the same conditions as control (**a**). Survival colonies could only be obtained from the transform manipulations with mutations of 0336 (pSS4) (**b**), NC2-1 (pHS58) (**c**), NC2-2 (pHS59) (**d**) and NC2-3 (pHS60) (**e**). More than two independent replications of this experiment were performed to ensure the reliabilities.

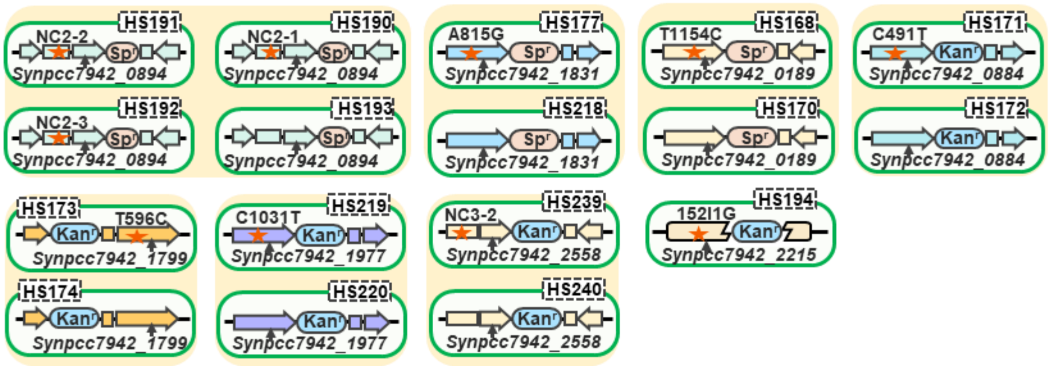

Supplementary Figure 11. Genotype diagrams of recombinant strains described in Fig 5a. strategy II. The recombinant strains were obtained by transforming the respective plasmids into the wildtype *Synechococcus* and selected utilizing the respective antibiotics.

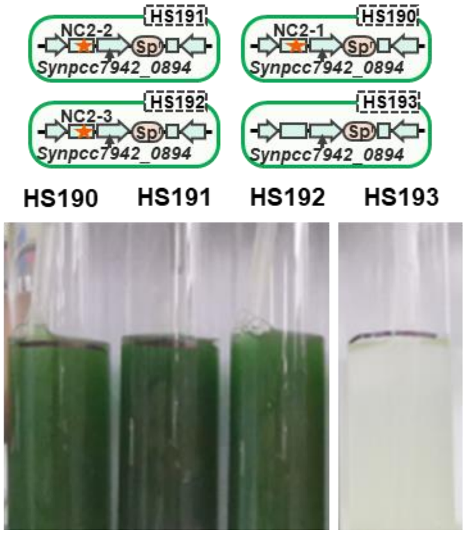

Supplementary Figure 12. Growths of the recombinant strains carrying NC2 mutations under HLHT conditions. HS190, HS191 and HS193 contains NC2-1, NC2-2, and NC2-3 as well as antibiotic resistance gene fragments, respectively. HS193 contains only a fragment of the antibiotic resistance gene. The recombinant strains were cultured under 42°C and 1500 μmol photons/m^2/s conditions and the cultures in column photobioreactors of the strains were photographed after 48 h of cultivation.. The recombinant strains were obtained by transforming the respective plasmids into the wildtype *Synechococcus* and selected utilizing the respective antibiotics. More than two independent replications of this experiment were performed to ensure the reliabilities.

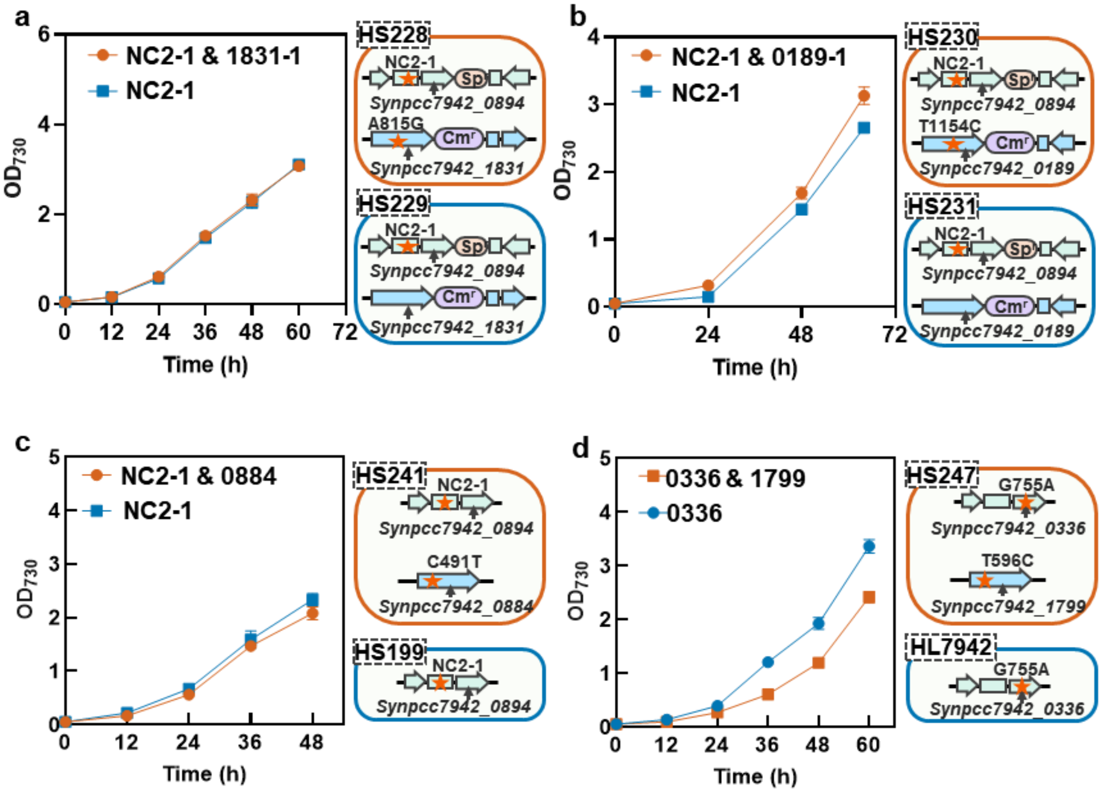

Supplementary Figure 13. Growths of the recombinant strains with different SNPs under NLNT conditions. a, The effects of the 1831-1 mutation and the NC2-1 mutation (in the strain HS228) on cell growths of the strain only carrying NC2-1 (HS229). **b,** The effects of the 0189-1 mutation and the NC2-1 mutation (in the strain HS230) on cell growths of the strain only carrying NC2-1 (HS231). **c,** The effects of the 0884 mutation and the NC2-1 mutation (in the strain HS241) on cell growths of the strain only carrying NC2-1 (HS199). **d,** The effects of the 1799 mutation and 0336 mutation (in the strain HS247) on cell growths of the strain only carry 0336 mutation (HL7942). NLNT conditions refer to 30°C and 500 μmol photons/m^2/s. More than two independent replications of each experiment were performed to ensure the reliabilities. Data are presented as mean values ± SD (n = 3 biological replicates). Source data are provided as a Source Data file.

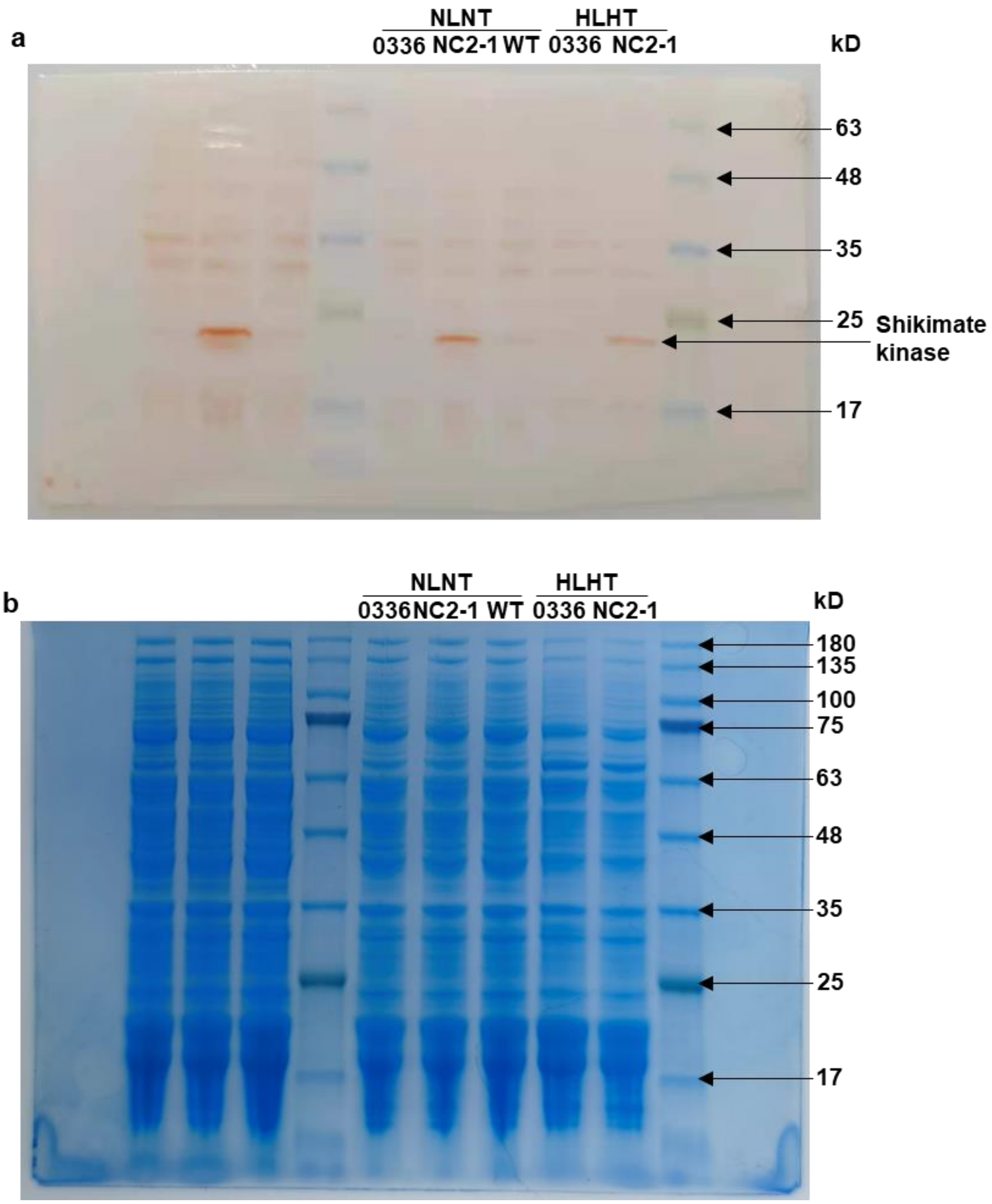

Supplementary Figure 14. Western blot analysis and SDS-PAGE of the *synpcc7942_0894* encoded shikimate kinase in HS199 strain. HL7942 strain carrying the 0336 mutation and showing HLHT was utilized as an additional control besides the WT to evaluate the effects of HLHT cultivation. Shown were the original images of western blot analysis (**a**) and SDS-PAGE (**b**) of the *synpcc7942_0894* encoded protein. The experiment was replicated more than twice to ensure its reliabilities.

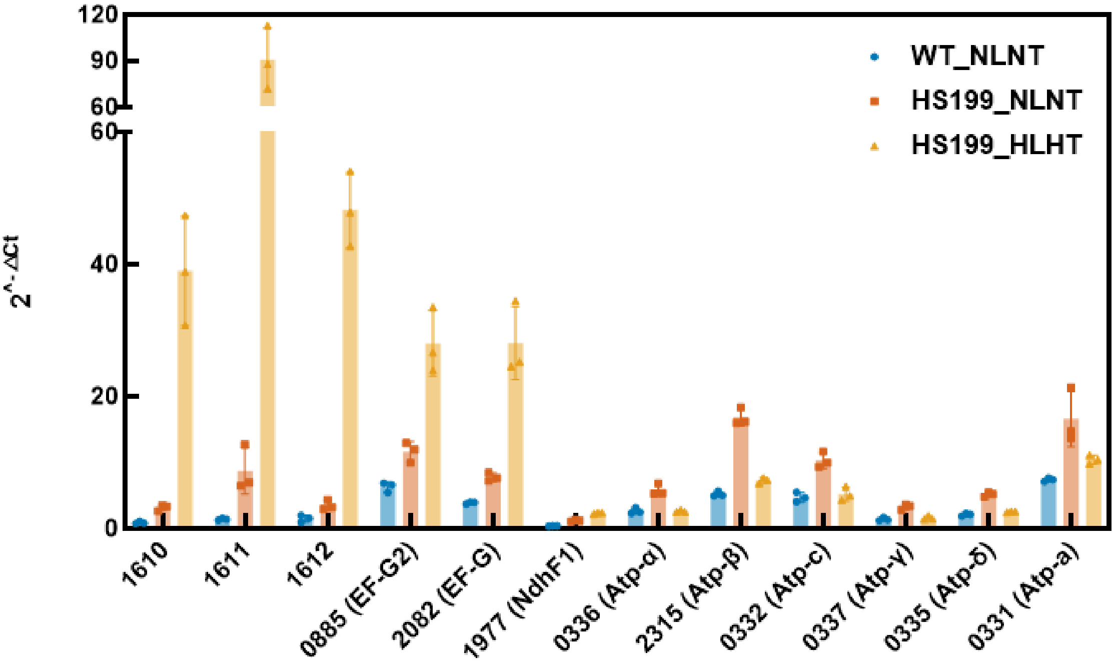

Supplementary Figure 15. Relative mRNA abundances of key genes involved in photosynthesis in HS199 and WT. The horizontal axis represents genes (1610 is the abbreviation of *synpcc7942_1610*, the others are named following the same principle). NLNT conditions refer to 30°C and 500 μmol photons/m^2/s, and HLHT conditions refer to 42°C and 1500 μmol photons/m^2/s. Data are presented as mean values ± SD (n = 3 biological replicates). More than two independent replications of this experiment were performed to ensure the reliabilities. Source data are provided as a Source Data file.

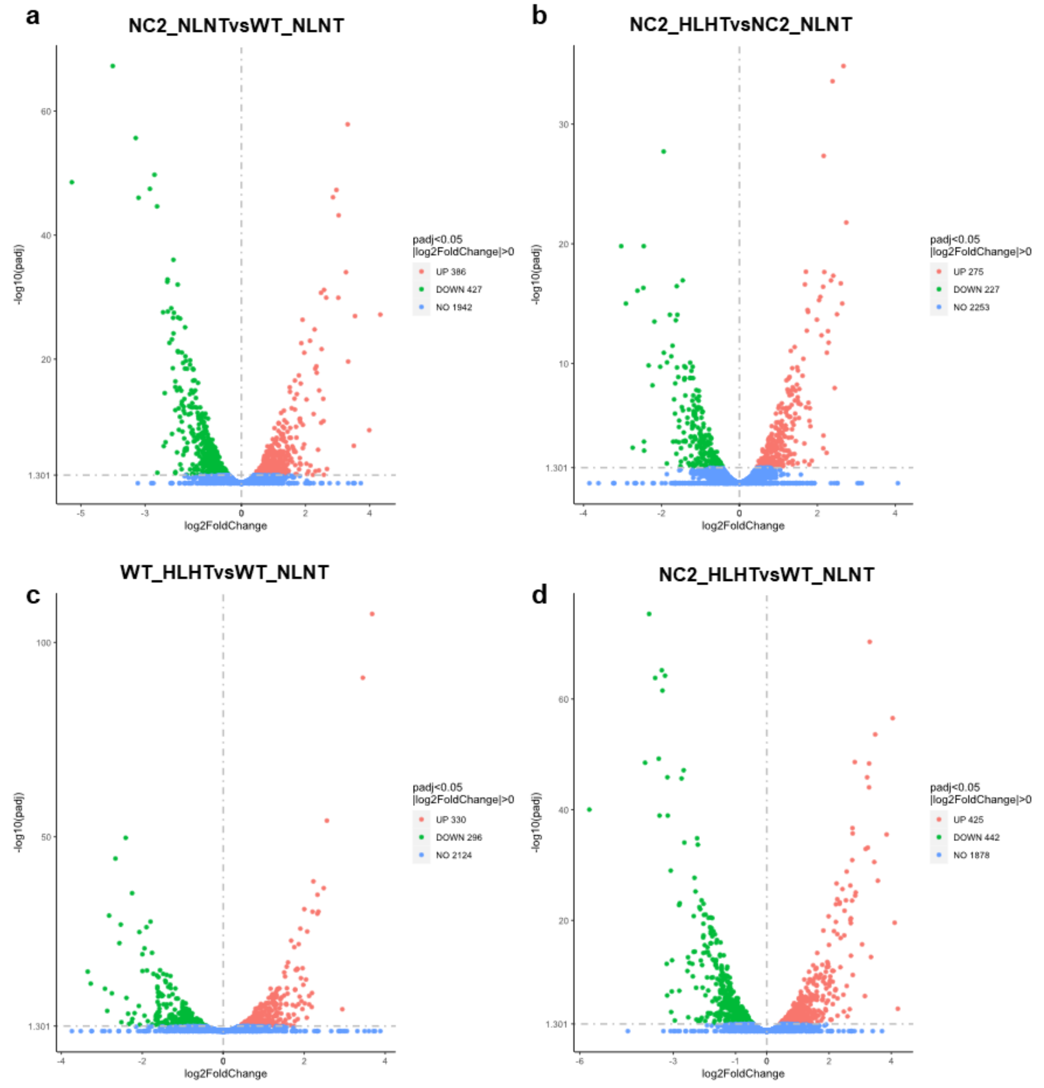

Supplementary Figure 16. Volcano plot of differentially expressed genes between different groups. The transcriptome of wildtype and HS199 was analyzed by RNA-seq under NLNT and HLHT conditions. For NLNT conditions, WT and HS199 were cultured at 30°C and 500 μmol photons/m^2/s to mid-exponential-phase, and then sampled and detected. The experimental groups were WT_NLNT and NC2_NLNT. For HLHT conditions, WT was cultured to the mid-exponential-phase at 30°C and 500 μmol photons/m^2/s, then treated at 42°C, 2000 μmol photons/m^2/s for 12 h, while HS199 was cultured to the mid-exponential-phase at 42°C, 2000 μmol photons/m^2/s, and then sampled and detected. The experimental groups were WT_HLHT and NC2_HLHT, the same below. Genes with an adjusted *P*-value (*Padj*) <0.05 were assigned as differentially expressed. Shown were the volcano plot in different comparisons of NC2_NLNTvsWT_NLNT (**a**), NC2_HLHTvsNC2_NLNT (**b**), WT_HLHTvsWT_NLNT (**c**), and NC2_vsWT_NLNT (**d**).

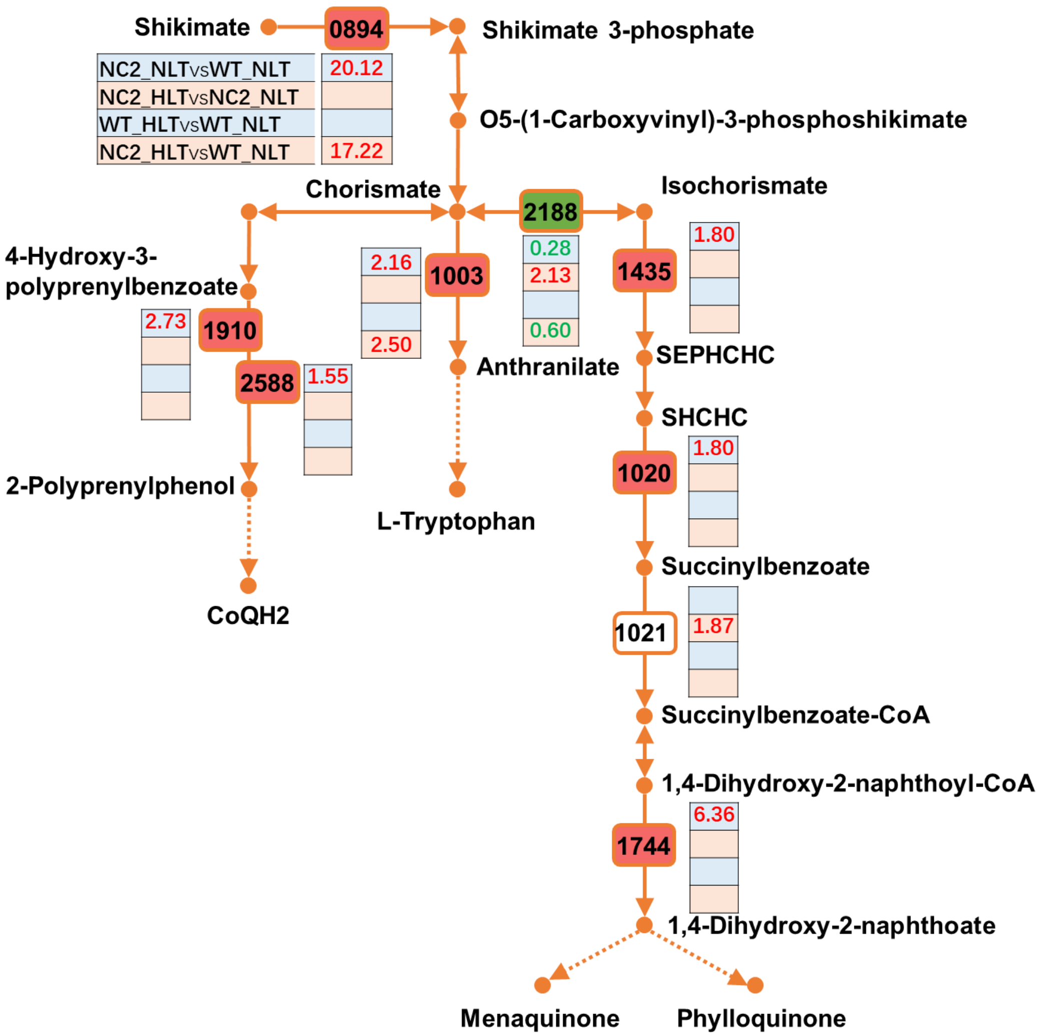

Supplementary Figure 17. NC2 mutation caused transcription changes of the genes involved in the pathways initialized by shikimate kinase. The connecting lines refer to the reaction and the arrows represent the reaction direction, while the RoundRect on it represents the enzyme and the number in it is the entry in KEGG of the corresponding coding genes (0894 is the abbreviation of *synpcc7942_0894*, the others are named following the same principle). The transcriptional fold difference in each comparison was listed nearby the enzymes, and the numbers from top to bottom correspond to the transcriptional fold changes of this gene in the four comparisons of NC2_NLNTvsWT_NLNT, NC2_HLHTvsNC2_NLNT, WT_HLHTvsWT_NLNT, and NC2_HLHTvsWT_NLNT in turn. The red RoundRect means the transcription of the enzyme was up-regulated in NC2_NLNT compared with WT_NLNT while the green RoundRect means the transcription of the enzyme was down-regulated in NC2_NLNT compared with WT_NLNT. CoQH2, Ubiquinol-n. SEPHCHC, 2-Succinyl-5-enolpyruvyl-6-hydroxy-3-cyclohexene-1-carboxylate. SHCHC, (1R,6R)-2-Succinyl-6-hydroxy-2,4-cyclohexadiene-1-carboxylate.

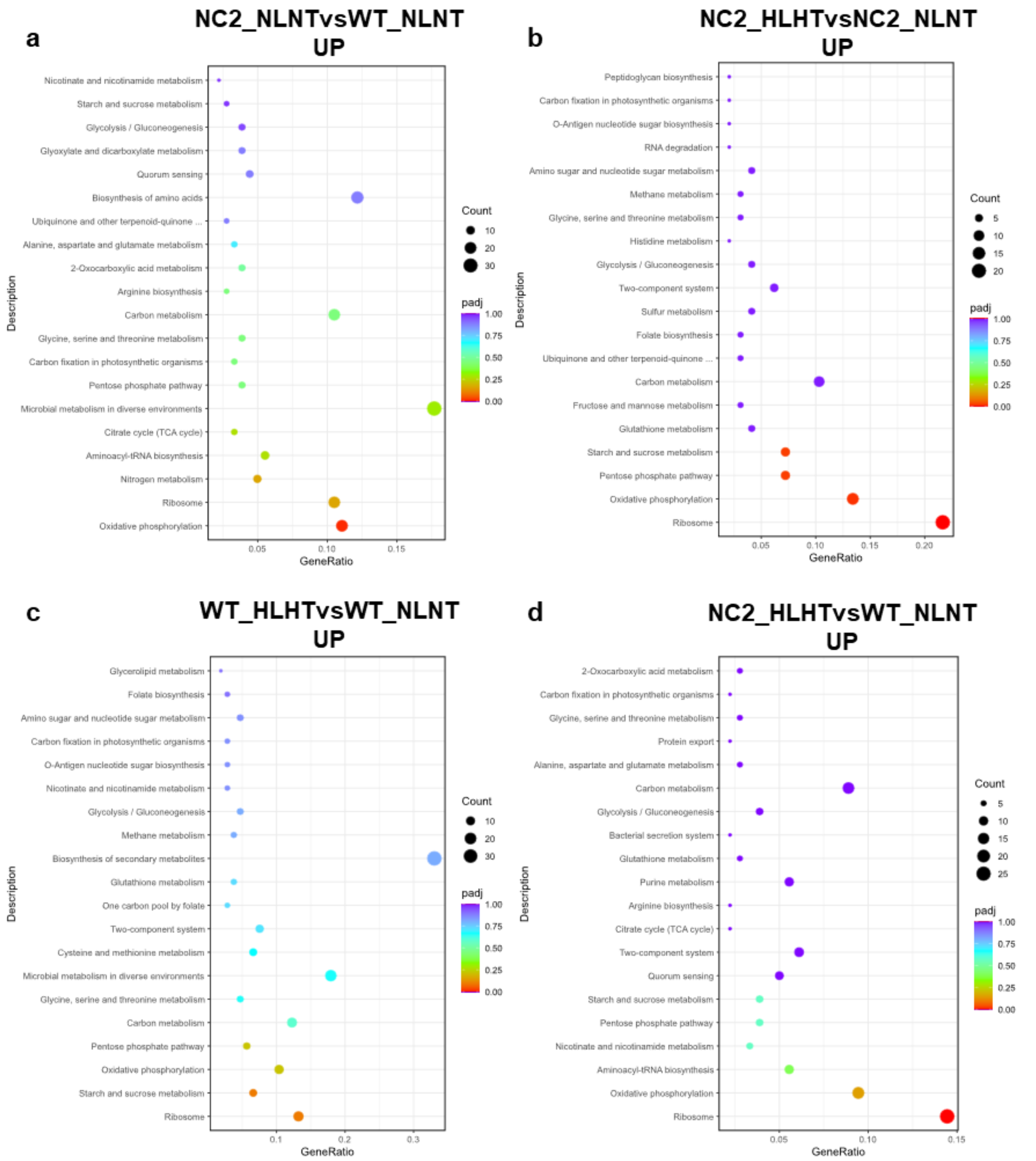

Supplementary Figure 18. KEGG enrichment of the up-regulated pathway between different groups. The dot size indicates the number of differentially expressed genes (DEGs) in certain pathways and the adjusted P-value was shown with colors. Shown were up-regulated KEGG pathways in different comparisons of NC2_NLNTvsWT_NLNT (**a**), NC2_HLHTvsNC2_NLNT (**b**), WT_HLHTvsWT_NLNT (**c**), and NC2_HLHTvsWT_NLNT (**d**).

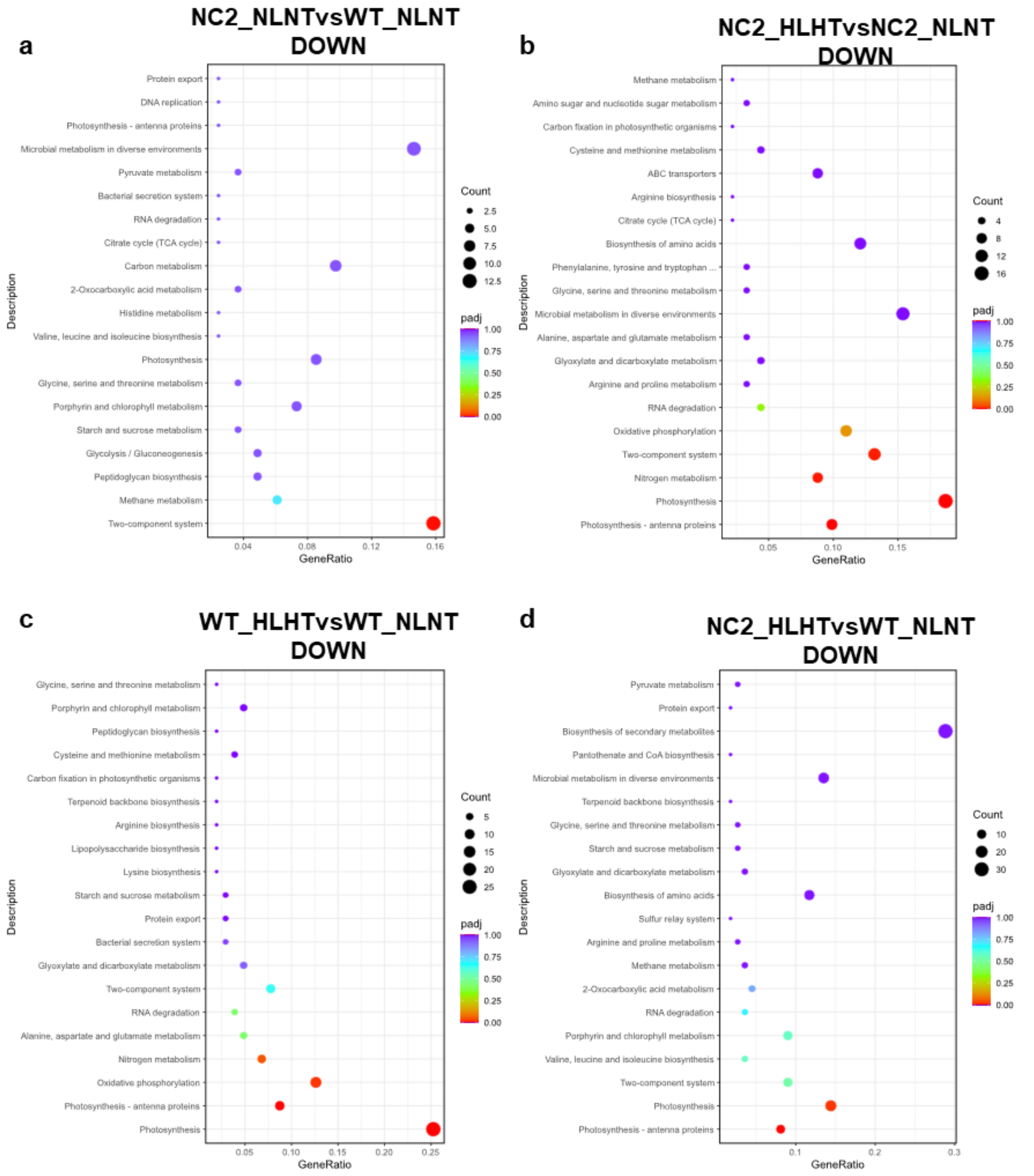

Supplementary Figure 19. KEGG enrichment of the down-regulated pathway between different groups. The dot size indicates the number of differentially expressed genes (DEGs) in certain pathways and the adjusted P-value was shown with colors. Shown were down-regulated KEGG pathways in different comparisons of NC2_NLNTvsWT_NLNT (**a**), NC2_HLHTvsNC2_NLNT (**b**), WT_HLHTvsWT_NLNT (**c**), and NC2_HLHTvsWT_NLNT (**d**).

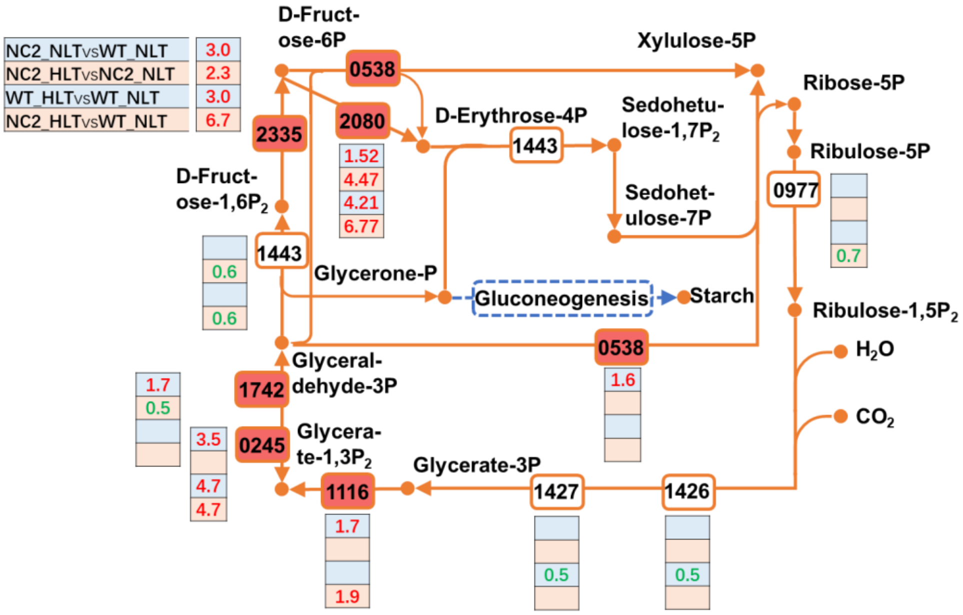

Supplementary Figure 20. NC2 mutation caused transcription changes of the genes involved in carbon fixation pathways. The connecting lines refer to the reaction and the arrows represent the reaction direction, while the RoundRect on it represents the enzyme and the number in it is the entry in KEGG of the corresponding coding genes (0538 is the abbreviation of *synpcc7942_0538*, the others are named following the same principle). The transcriptional fold difference in each comparison was listed nearby the enzymes, and the numbers from top to bottom correspond to the transcriptional fold changes of this gene in the four comparisons of NC2_NLNTvsWT_NLNT, NC2_HLHTvsNC2_NLNT, WT_HLHTvsWT_NLNT, and NC2_HLHTvsWT_NLNT in turn. The red RoundRect means the transcription of the enzyme was up-regulated in NC2_NLNT compared with WT_NLNT while the green RoundRect means the transcription of the enzyme was down-regulated in NC2_NLNT compared with WT_NLNT. P in each chemical refers to phosphate.

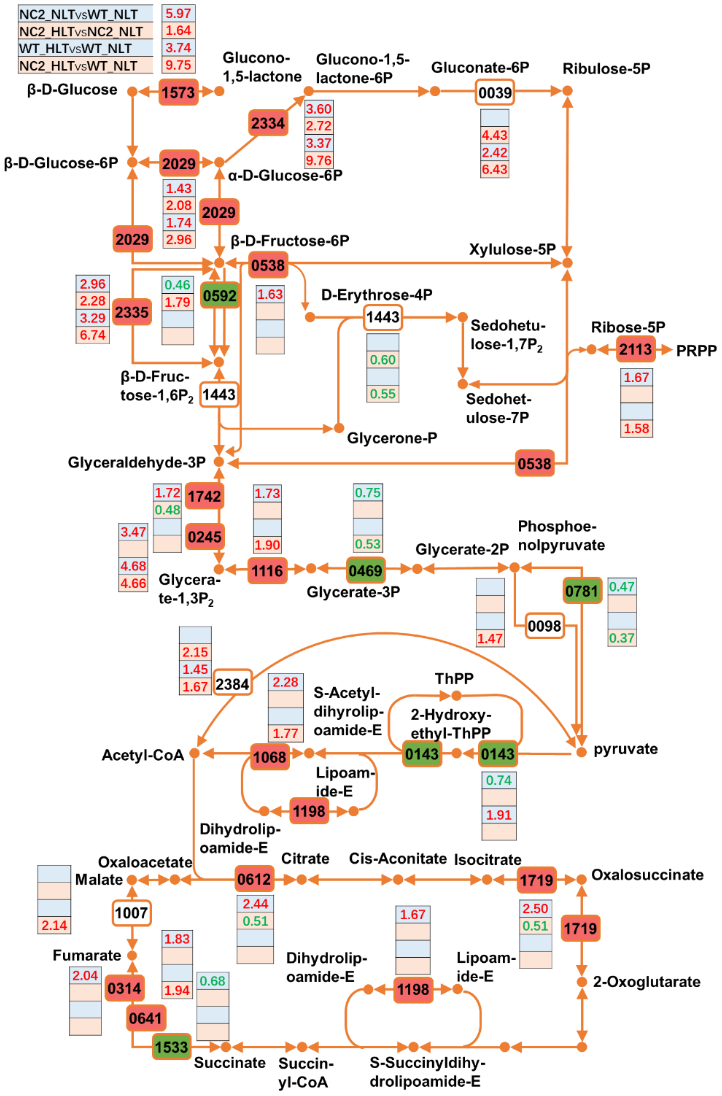

Supplementary Figure 21. NC2 mutation caused transcription changes of the genes involved in carbon metabolism pathways. The connecting lines refer to the reaction and the arrows represent the reaction direction, while the RoundRect on it represents the enzyme and the number in it is the entry in KEGG of the corresponding coding genes (1573 is the abbreviation of *synpcc7942_1573*, the others are named following the same principle). The transcriptional fold difference in each comparison was listed nearby the enzymes, and the numbers from top to bottom correspond to the transcriptional fold changes of this gene in the four comparisons of NC2_NLNTvsWT_NLNT, NC2_HLHTvsNC2_NLNT, WT_HLHTvsWT_NLNT, and NC2_HLHTvsWT_NLNT in turn. The red RoundRect means the transcription of the enzyme was up-regulated in NC2_NLNT compared with WT_NLNT while the green RoundRect means the transcription of the enzyme was down-regulated in NC2_NLNT compared with WT_NLNT. PRPP, 5-Phospho-alpha-D-ribose 1-diphosphate. P in each chemical refers to phosphate.

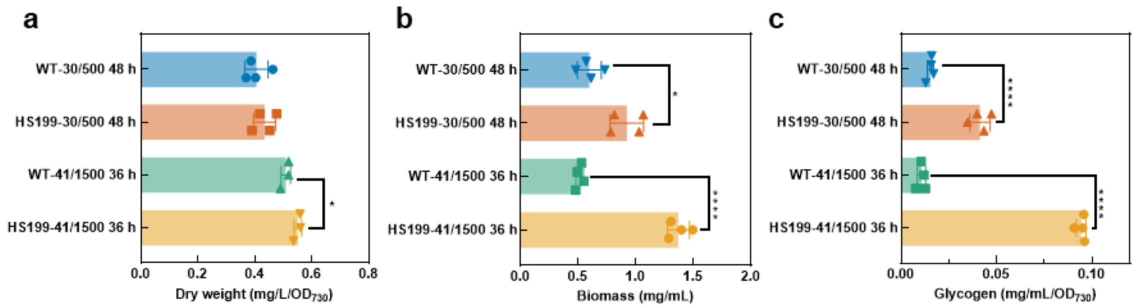

Supplementary Figure 22. Physiological parameters of WT and HS199 under NLNT and HLHT conditions. Dry weight (a), biomass (b) and glycogen content (c) of WT and HS199 at 30°C and 500 μmol photons/m^2/s (30/500, NLNT) for 48 h and 41°C and 1500 μmol photons/m^2/s (41/1500, HLHT) for 36 h. Data are presented as mean values ± SD. n = 4 biological replicates except that n = 3 in WT-41/1500 36 h and HS199-41/1500 36 h of **a**. Statistical analysis was performed using two-tailed unpaired Student's *t*-test (*$p < 0.05$, ****$p < 0.0001$). The p value in **a** is 0.0265. The p values in **b** and **c** are $p = 0.0110$, $p < 0.0001$ and $p < 0.0001$, $p < 0.0001$ (from left to right). Each experiment was replicated more than twice to ensure their reliabilities. Source data are provided as a Source Data file.

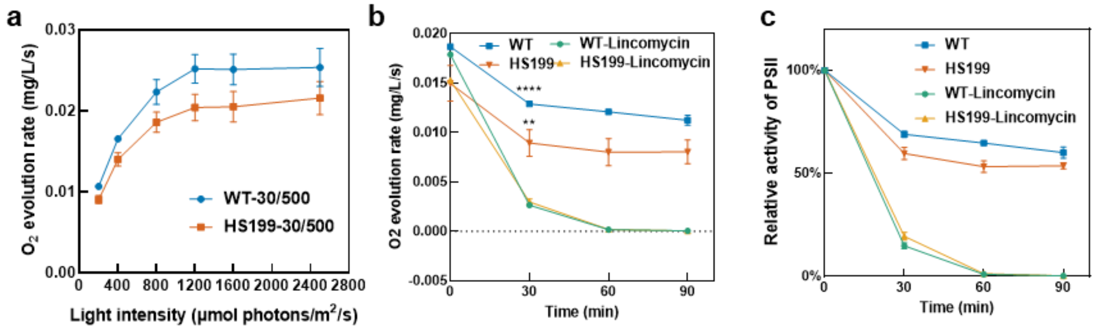

Supplementary Figure 23. PSII-mediated O_2 evolution of WT and HS199. a. PSII-mediated O_2 evolution of WT and HS199 under 30°C and 500 μmol photons/m^2/s (30/500). **b** and **c**. PSII-mediated O_2 evolution **(b)** and the PSII activity **(c)** of WT and HS199 during exposure to HLHT stress (42°C and 2500 μmol photons/m^2/s) with or without lincomycin. PSII-mediated O_2 evolution rate during exposure to HLHT stress was normalized by setting the PSII-mediated O_2 evolution rate under NLNT conditions to 100%. The PSII-mediated O_2 evolution of WT and HS199 after 30 min HLHT treatment and before treatment were significantly analyzed using two-tailed unpaired Student's *t*-test (***p* < 0.01, *****p* < 0.0001). The *p* values are *p* < 0.0001 and *p* = 0.0098 (from up to down). Data are presented as mean values ± SD (n = 3 biological replicates). Each experiment was replicated more than twice to ensure their reliabilities. Source data are provided as a Source Data file.

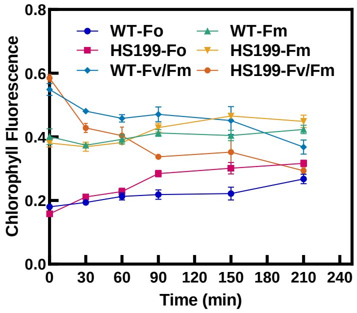

Supplementary Figure 24. The *Fv/Fm* of WT and HS199 after exposed to HLHT conditions. HLHT conditions refers to 42°C and 2500 μmol photons/m^2/s. Data are presented as mean values ± SD (n = 3 biological replicates except the *Fo, Fm, Fv/Fm* of HS199 at 30 min which includes 2 biological replicates). The experiment was replicated more than twice to ensure their reliabilities. Source data are provided as a Source Data file.

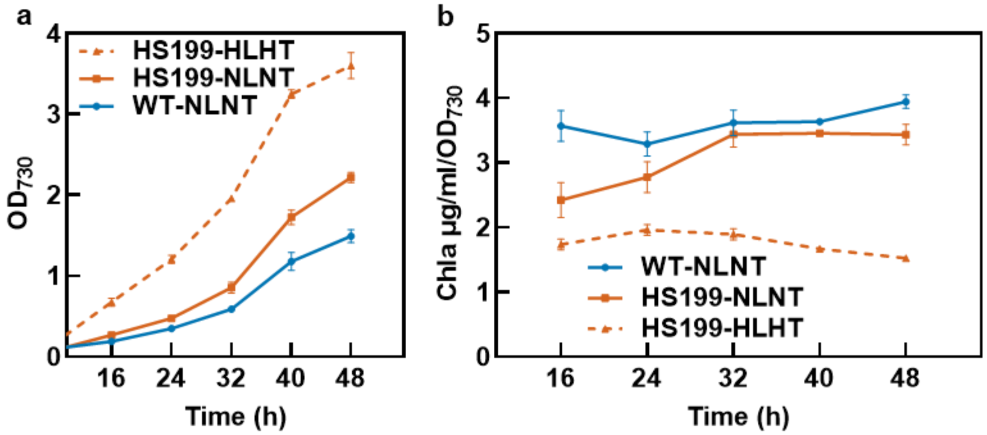

Supplementary Figure 25. Cell growths and pigment content of WT and HS199. Cell growths (**a**) and pigment content (**b**) of WT and HS199 were monitored during the cultivation under normal temperature and light intensities (30°C and 500 μmol photons/m^2/s, NLNT) and high temperature and high light intensities (42°C and 2000 μmol photons/m^2/s, HLHT). More than two independent replications of this experiment were performed to ensure the reliabilities. Data are presented as mean values ± SD (n = 3 biological replicates). Source data are provided as a Source Data file.

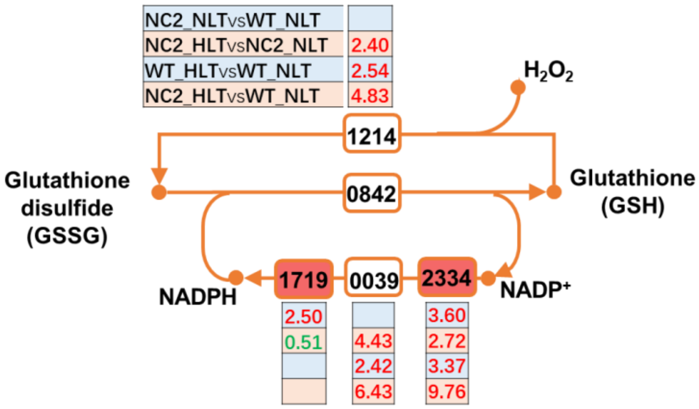

Supplementary Figure 26. Fold changes in transcript abundance of genes in transformation between glutathione and glutathione disulfide in different comparisons. The connecting lines refer to the reaction and the arrows represent the reaction direction, while the RoundRect on it represents the enzyme and the number in it is the entry in KEGG of the corresponding coding genes (1719 is the abbreviation of *synpcc7942_1719*, the others are named following the same principle). The transcriptional fold difference in each comparison was listed nearby the enzymes, and the numbers from top to bottom correspond to the transcriptional fold changes of this gene in the four comparisons of NC2_NLNTvsWT_NLNT, NC2_HLHTvsNC2_NLNT, WT_HLHTvsWT_NLNT, and NC2_HLHTvsWT_NLNT in turn. The red RoundRect means the transcription of the enzyme was up-regulated in NC2_NLNT compared with WT_NLNT.

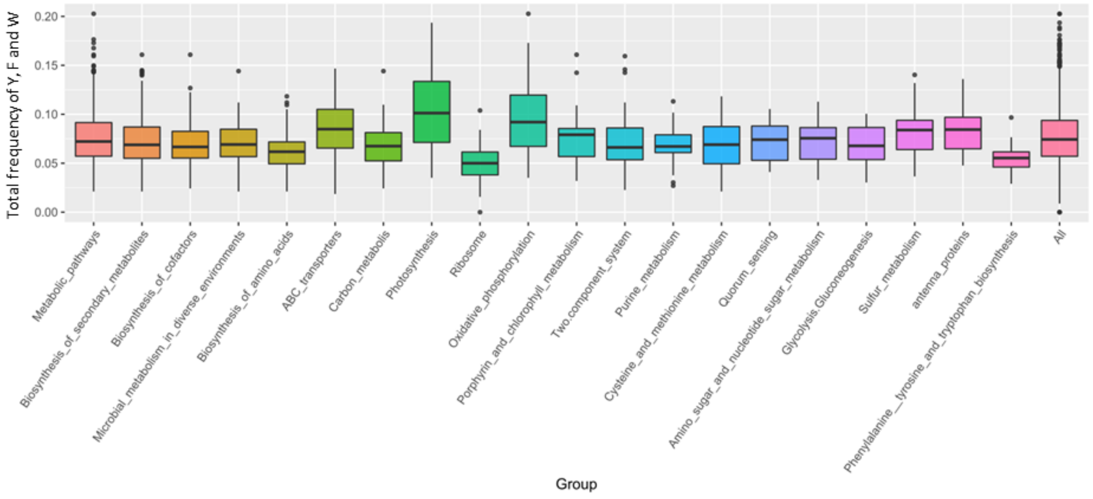

Supplementary Figure 27. The top 20 KEGG pathways with the highest frequency of the three aromatic amino acids in proteins. The three aromatic amino acids include Y (tyrosine), F (phenylalanine), and W (tryptophan). The proteome of *Synechococcus elongatus* PCC 7942 was downloaded from UniProt (20210730, UP000002717_1140) and 2657 proteins were involved. Total frequency of Y, F, and W in each protein was calculated using R, and the result was listed in Dataset S5. Proteins were enriched into the corresponding pathway via KEGG. Boxplot is traditional boxplot with median, and upper and lower quartiles. The sample size n was consistent with the number of proteins in each pathway (n = 558, 252, 129, 115, 92, 68, 68, 60, 53, 46, 45, 41, 38, 33, 29, 28, 27, 25, 14, 19, and 2657; from left to right). Source data are provided as a Source Data file.

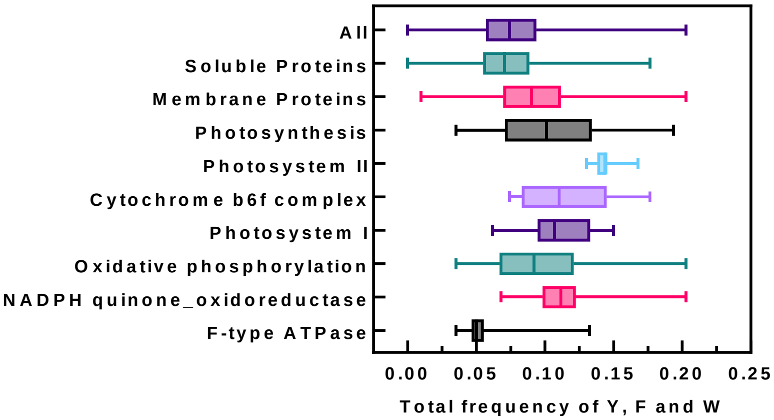

Supplementary Figure 28. Total frequencies of the three aromatic amino acids in proteins belonging to different classes. The three aromatic amino acids include Y (tyrosine), F (phenylalanine), and W (tryptophan). Boxplot is traditional boxplot with median, and upper and lower quartiles. The sample size n was consistent with the number of proteins in each pathway (n = 2657, 2097, 560, 60, 8, 8, 6, 46, 20, and 9; from up to down). Source data are provided as a Source Data file.

Supplementary Table 1. Selection of putative genes in *Synechococcus* to regulate DNA replication fidelity.

**Entry**	**Definition**	**DNA fidelity mechanism**	**Gene name**	**Manipulation**
Synpcc7942_0563	Excinuclease ABC subunit B	Nucleotide excision repair	*uvrB*	knock out
Synpcc7942_1945	Excinuclease ABC subunit C	Nucleotide excision repair	*uvrC*	knock out
Synpcc7942_1780	DNA mismatch repair protein MutL	Mismatch repair	*mutL*	knock out
Synpcc7942_2247	DNA mismatch repair protein MutS	Mismatch repair	*mutS*	knock out
Synpcc7942_1790	DNA adenine methylase	Mismatch repair	*dam*	knock out
Synpcc7942_0673	A/G-specific DNA-adenine glycosylase	Base excision repair	*mutY*	knock out
Synpcc7942_1323	Formamidopyrimidine-DNA glycosylase	Base excision repair	*mutM*	knock out
Synpcc7942_2607	Exodeoxyribonuclease III	Base excision repair	*xthA*	knock out
Synpcc7942_0251	DNA polymerase III subunit epsilon	Proofreading	*dnaQ*	overexpression; D26A, E28A
Synpcc7942_1550	DNA polymerase V	Translesion DNA synthesis	*umuC*	overexpression
Synpcc7942_1549	DNA polymerase V	Translesion DNA synthesis	*umuD*	overexpression
Synpcc7942_0348	Recombination protein RecA	Homologous recombination; translesion DNA synthesis	*recA*	overexpression
Synpcc7942_1779	DNA repair protein RecN	Homologous recombination; translesion DNA synthesis	*recN*	overexpression

Supplementary Table 2. Overview of the SNPs in the non-coding region in HLHT-tolerant evolved *Synechococcus* strains.

Genes nearby the mutation	Encoded proteins	Pathway	Non-coding regions[a]	SNPs	Code[b]
Synpcc7942_0894	Shikimate kinase	Phenylalanine, tyrosine and tryptophan biosynthesis	NC2	G905452A	NC2-1
Synpcc7942_0895	Conserved hypothetical protein			C905470T	NC2-2
				G905445A	NC2-3
Synpcc7942_2557	NAD-reducing hydrogenase HoxS gamma subunit	Oxidative phosphorylation	NC3	T2636387G	NC3-1
Synpcc7942_2558	Cysteine desulfurase NifS	Thiamine metabolism		G2636431A	NC3-2
Synpcc7942_2368	Secretion protein HlyD		NC4	T2433814C	NC4
Synpcc7942_2367	Conserved hypothetical protein				
Synpcc7942_2355	Conserved hypothetical protein		NC5	T2422163C	NC5
Synpcc7942_2356	Transcriptional regulator, Crp/Fnr family				
Synpcc7942_0313	Conserved hypothetical protein	Oxidative phosphorylation	NC6	A312315G	NC6
Synpcc7942_0314	Succinate dehydrogenase subunit C				

a. The non-coding intergenic regions where the SNPs were located were termed NC2-NC6.

b. The mutations including SNPs and InDels were coded as described in Fig. 4, the same below.

Supplementary Table 3. Overview of the SNPs in the CDS region in HLHT-tolerant evolved *Synechococcus* strains.

Genes	Encoded proteins	Pathway	Mutation	SNPs	Code
Synpcc7942_0336	ATP synthase F1, alpha subunit	Oxidative phosphorylation	C252Y	G331632A	0336
Synpcc7942_1799	Hydrogenase expression/formation protein HypE	Hydrogenase maturation factor	L199P	T1869535C	1799
Synpcc7942_1831	IMP dehydrogenase related 2	Purine metabolism	K272R	T1900205C	1831-1
			Y248C	T1900277C	1831-2
			C183Y	C1900472T	1831-3
			A53S	C1900862A	1831-4
Synpcc7942_0189	GMP synthase	Purine metabolism	V385A	A188105G	0189-1
			G371D	C188147T	0189-2
Synpcc7942_0884	Translation elongation factor 1A (EF-1A/EF-Tu)	Translation factors	P164L	G890436A	0884
Synpcc7942_2524	Trigger factor	Protein processing	A390A	A2606447G	2524
Synpcc7942_2087	Imidazole glycerol phosphate synthase subunit hisF	Histidine metabolism	A208T	G2167770A	2087
Synpcc7942_1439	Proton-translocating NADH-quinone oxidoreductase, chain M	Oxidative phosphorylation	Nonsense mutation	C1494008T	1439
Synpcc7942_1589	Putative modulator of DNA gyrase	Peptidases and inhibitors	A337V	G1656261A	1589
Synpcc7942_2110	Methionyl-tRNA formyltransferase	Aminoacyl-tRNA biosynthesis	T146T	C2191463T	2110
Synpcc7942_1377	Metal dependent phosphohydrolase	Purine metabolism	E404G	A1418750G	1377
Synpcc7942_1269	Magnesium transporter	Transporters	A119V	C1293897T	1269
Synpcc7942_1977	Proton-translocating NADH-quinone oxidoreductase, chain L	Oxidative phosphorylation	A338V	G2048552A	1977
Synpcc7942_0853	Aminotransferase	Lysine biosynthesis	N186S	A849426G	0853
Synpcc7942_2369	Hydrophobe/amphiphile efflux-1 HAE1	Signaling and cellular processes	G182G	T2435586C	2369
Synpcc7942_0652	Lycopene cyclase CruP	Carotenoid biosynthesis	I143T	A648286G	0652
Synpcc7942_0808	HAD-superfamily hydrolase subfamily IIB	Starch and sucrose metabolism	T579T	T801244C	0808
Synpcc7942_1816	Periplasmic sensor hybrid histidine kinase		P340P	A1890358G	1816
Synpcc7942_0813	Histidyl-tRNA synthetase 2, putative	Histidine metabolism	V51A	T807990C	0813
Synpcc7942_0455	Queuine tRNA-ribosyltransferase	Transfer RNA biogenesis	Y327C	A444980G	0455
Synpcc7942_2252	Phosphoenolpyruvate carboxylase	Pyruvate metabolism	D664G	T2319990C	2252
Synpcc7942_0106	Probable nicotinate-nucleotide adenylyltransferase	Nicotinate and nicotinamide metabolism	V85A	A106699G	0106
Synpcc7942_2527	Esterase-like		L120P	T2609233C	2527
Synpcc7942_0973	UDP-glucose 6-dehydrogenase	Pentose and glucuronate interconversions	N172S	T980416C	0973
Synpcc7942_1979	Membrane protein-like		V202A	A2050132G	1979
Synpcc7942_0858	Methyl-accepting chemotaxis protein PixJ	Two-component system	A951A	A858745G	0858

Supplementary Table 4. Overview of the InDels in HLHT-tolerant evolved *Synechococcus* strains.

Genes	Encoded proteins	Pathway	InDels	Code
Synpcc7942_0156	Phosphoglucomutase	Pentose phosphate pathway	158234 D1C	0156
Synpcc7942_2215	Large subunit ribosomal protein L15	Translation	2291255 D1C	2215-1
			2291255 I1C	2215-2
Synpcc7942_0563	Excinuclease ABC subunit B	Nucleotide excision repair	543656 I1T	0563
Synpcc7942_0595	Hypothetical protein		583303 D1G	0595
Synpcc7942_2241	Conserved hypothetical protein		2306808 I1G	2241
Synpcc7942_0659	Rad3-related DNA helicases-like		654969 D1G	0659
Synpcc7942_B2622	Probable chromate transport transmembrane protein	Signaling and cellular processes	14172 D1T	B2622

Supplementary Table 5. DEGs in transcription factors between different groups.

		Foldchanges in different comparisons				
**Genes**	**Proteins**	**NC2_NTNLvsWT_NTNL**	**NC2_HTHLvsNC2_NTNL**	**WT_HTHLvsWT_NTNL**	**NC2_HTHLvsWT_NTNL**	**NC2_HTHLvsWT_HTHL**
Synpcc7942_0649	SigA	1.52				
Synpcc7942_0672	SigD					
Synpcc7942_1746	SigB	0.50		0.64	0.36	0.56
Synpcc7942_1849	SigC	1.91	6.22	10.91	11.85	
Synpcc7942_1557	SigM	0.27		0.46	0.30	0.65
Synpcc7942_0569	SigM		0.37	0.44	0.32	
Synpcc7942_1510	SigF	0.69	0.56	0.72	0.39	0.54
Synpcc7942_1784	SigF2		0.38	0.67	0.36	0.53
Synpcc7942_1923	SigG			1.49		0.73
