# Supplementary material for: Engineered hypermutation adapts cyanobacterial photosynthesis to combined high light and high temperature stress
Source: Nat Commun. 2023 Mar 4;14:1238. doi: 10.1038/s41467-023-36964-5 (PMC9985602; doi:10.1038/s41467-023-36964-5)
Supplement: Supplementary file 3 — Description of Additional Supplementary Files [file 41467_2023_36964_MOESM3_ESM.pdf]

Description of Additional Supplementary Files

File Name: **Supplementary Data 1**
Description: The input data of variant analysis.

File Name: **Supplementary Data 2**
Description: Plasmids used in this study.

File Name: **Supplementary Data 3**
Description: The RNA seq data of NC2_NLNTvsWT_NLNT.

File Name: **Supplementary Data 4**
Description: Protein abundance determined by PRM.

File Name: **Supplementary Data 5**
Description: The RNA seq data of NC2_HLHTvsNC2_NLNT.

File Name: **Supplementary Data 6**
Description: The RNA seq data of NC2_HLHTvsWT_NLNT.

File Name: **Supplementary Data 7**
Description: The RNA seq data of WT_HLHTvsWT_NLNT.

File Name: **Supplementary Data 8**
Description: Total frequencies of Y, F, and W of of proteins in *Synechococcus.*

File Name: **Supplementary Data 9**
Description: Total frequencies of Y, F, and W of membrane proteins.

File Name: **Supplementary Data 10**
Description: Total frequencies of Y, F, and W of soluble proteins.

File Name: **Supplementary Data 11**
Description: Total frequencies of Y, F, and W of proteins in Photosynthesis and Oxidative Phosphorylation.

File Name: **Supplementary Data 12**
Description: Cyanobacterial strains used in this study.

File Name: **Supplementary Data 13**
Description: Primers used in this study.
