# Supplementary material for: Engineered hypermutation adapts cyanobacterial photosynthesis to combined high light and high temperature stress
Source: Nat Commun. 2023 Mar 4;14:1238. doi: 10.1038/s41467-023-36964-5 (PMC9985602; doi:10.1038/s41467-023-36964-5)
Supplement: Supplementary file 17 — Reporting Summary [file 41467_2023_36964_MOESM17_ESM.pdf]

nature portfolio

Corresponding author(s): Xuefeng Lu

Last updated by author(s): Feb 22, 2023

Reporting Summary

Nature Portfolio wishes to improve the reproducibility of the work that we publish. This form provides structure for consistency and transparency in reporting. For further information on Nature Portfolio policies, see our Editorial Policies and the Editorial Policy Checklist.

Statistics

For all statistical analyses, confirm that the following items are present in the figure legend, table legend, main text, or Methods section.

n/a	Confirmed	
☐	☒	The exact sample size (*n*) for each experimental group/condition, given as a discrete number and unit of measurement
☐	☒	A statement on whether measurements were taken from distinct samples or whether the same sample was measured repeatedly
☐	☒	The statistical test(s) used AND whether they are one- or two-sided<br>*Only common tests should be described solely by name; describe more complex techniques in the Methods section.*
☒	☐	A description of all covariates tested
☐	☒	A description of any assumptions or corrections, such as tests of normality and adjustment for multiple comparisons
☐	☒	A full description of the statistical parameters including central tendency (e.g. means) or other basic estimates (e.g. regression coefficient) AND variation (e.g. standard deviation) or associated estimates of uncertainty (e.g. confidence intervals)
☐	☒	For null hypothesis testing, the test statistic (e.g. *F*, *t*, *r*) with confidence intervals, effect sizes, degrees of freedom and *P* value noted<br>*Give P values as exact values whenever suitable.*
☒	☐	For Bayesian analysis, information on the choice of priors and Markov chain Monte Carlo settings
☒	☐	For hierarchical and complex designs, identification of the appropriate level for tests and full reporting of outcomes
☒	☐	Estimates of effect sizes (e.g. Cohen's *d*, Pearson's *r*), indicating how they were calculated

Our web collection on statistics for biologists contains articles on many of the points above.

Software and code

Policy information about availability of computer code

Data collection	1. ImageJ 1.52a was used to count the rifampicin-tolerant colonies in mutation rate evaluation processes.<br>2. LightCycler 480 software 1.5 was used for RT-qPCR.<br>3. Dual PAM v1.19 was used for room temperature fluorescence kinetics and the determination of P700 kinetics.<br>4. YZQ-201A13 was used for the determination of photosynthetic O2 evolution and dark respiration.
Data analysis	1. For the whole-genome re-sequencing, the reference sequence (Synechococcus elongatus PCC 7942, FACHB-805) was obtained from GenBank for read mapping using the BWA software (V0.7.8). SAMTOOLS (v0.1.18) was used to detect individual SNPs and the InDels of small fragments (<50 bp), as well as to analyze the variation in SNP/InDel in the functional regions of the genome.<br>2. Code used for hierarchical clustering in variant analysis is available on Github at https://github.com/jibeilindong/Drawtree/blob/main/drawtree.r.<br>3. For RNA-seq analysis, The reference genome and gene model annotation files (Synechococcus elongatus PCC 7942 and FACHB-805) were obtained from GenBank for read mapping using Bowtie2 (v2.3.4.3). HTSeq (v0.9.1) was used to count the read numbers mapped to each gene, and then the FPKM of each gene was calculated. Differential expression analysis of the two groups (three biological replicates per condition) was performed using the DESeq R package (v1.20).<br>4. Code used for analyzing the frequency of tyrosine, phenylalanine, and tryptophan in proteins is available on https://github.com/jibeilindong/Drawtree/blob/main/AA_frequency.R.

For manuscripts utilizing custom algorithms or software that are central to the research but not yet described in published literature, software must be made available to editors and reviewers. We strongly encourage code deposition in a community repository (e.g. GitHub). See the Nature Portfolio guidelines for submitting code & software for further information.

Data

Policy information about availability of data

All manuscripts must include a data availability statement. This statement should provide the following information, where applicable:

- Accession codes, unique identifiers, or web links for publicly available datasets
- A description of any restrictions on data availability
- For clinical datasets or third party data, please ensure that the statement adheres to our policy

Source data are provided as a Source Data file. Raw data for Whole genome re-sequencing is available through NCBI under accession number PRJNA846529 [https://www.ncbi.nlm.nih.gov/bioproject/PRJNA846529]. Raw data for RNA-seq analysis is available through NCBI under accession number PRJNA847037 [https://www.ncbi.nlm.nih.gov/bioproject/?term=PRJNA847037). The reference genome and gene model annotation files (Synechococcus elongatus PCC 7942 and FACHB-805] for whole genome re-sequencing and RNA-seq were obtained from GenBank [https://ftp.ncbi.nlm.nih.gov/genomes/all/GCA/000/012/525/GCA_000012525.1_ASM1252v1/].
Code used for hierarchical clustering in variant analysis is available on Github at https://github.com/jibeilindong/Drawtree/blob/main/drawtree.r. Code used for analyzing the frequency of tyrosine, phenylalanine, and tryptophan in proteins is available on Github at https://github.com/jibeilindong/Drawtree/blob/main/AA_frequency.R.

Human research participants

Policy information about studies involving human research participants and Sex and Gender in Research.

Reporting on sex and gender	N/A
Population characteristics	N/A
Recruitment	N/A
Ethics oversight	N/A

Note that full information on the approval of the study protocol must also be provided in the manuscript.

Field-specific reporting

Please select the one below that is the best fit for your research. If you are not sure, read the appropriate sections before making your selection.

☒ Life sciences ☐ Behavioural & social sciences ☐ Ecological, evolutionary & environmental sciences

For a reference copy of the document with all sections, see nature.com/documents/nr-reporting-summary-flat.pdf

Life sciences study design

All studies must disclose on these points even when the disclosure is negative.

Sample size	No statistical method was used to determine sample size. Sample size was chosen based on previous experience and standards in the field. The sample size has been shown in each figure legend and methods, and at least three biological replicates were used.
Data exclusions	No data were excluded.
Replication	Each experiment was replicated several times independently and all attempts at replication were successful.
Randomization	Randomization was irrelevant because our study did not involve assigning samples to experimental groups.
Blinding	The group allocation was not involved in our study. So investigators were not blind to the group allocation. Investigators were not blind during acquisition and analysis as well. Because the collected data was the result of quantitative data detected by instruments and was generally not affected by subjective factors.

Reporting for specific materials, systems and methods

We require information from authors about some types of materials, experimental systems and methods used in many studies. Here, indicate whether each material, system or method listed is relevant to your study. If you are not sure if a list item applies to your research, read the appropriate section before selecting a response.

Materials & experimental systems

n/a	Involved in the study	
☐	☒	Antibodies
☒	☐	Eukaryotic cell lines
☒	☐	Palaeontology and archaeology
☒	☐	Animals and other organisms
☒	☐	Clinical data
☒	☐	Dual use research of concern

Methods

n/a	Involved in the study	
☒	☐	ChIP-seq
☒	☐	Flow cytometry
☒	☐	MRI-based neuroimaging

Antibodies

Antibodies used

1. Rabbit anti-protein Synechococcus shikimate kinase antibody (no. 21000874A-090221-A01; Atagenix, 1:1000)
2. Goat Anti-Rabbit lgG (HRP) (no. SE134; Solarbio, 1:2000)

Validation

The rabbit polyclonal primary antibody against the shikimate kinase of Synechococcus was prepared and purchased from Atagenix Technology Co., Ltd (Wuhan, China). The primary antibody was verified by indirect ELISA and western blot. The specificity of Goat Anti-Rabbit lgG (HRP) (no. SE134; Solarbio) was determined by Solarbio [https://solarbio.com/search.php?category=22&keywords=SE134&imageField=Search].
